# Improved Resolution Optical Time Stretch Imaging Based on High Efficiency In-Fiber Diffraction

**DOI:** 10.1038/s41598-017-18920-8

**Published:** 2018-01-12

**Authors:** Guoqing Wang, Zhijun Yan, Lei Yang, Lin Zhang, Chao Wang

**Affiliations:** 1 School of Engineering and Digital Arts, University of Kent, Canterbury, United Kingdom CT2 7NT; 2 School of Optical and Electronic Information (SOEI), Next Generation Internet Access National Engineering Laboratory (NGIAS), Huazhong University of Science and Technology, Wuhan, 430074 China; 3 Aston Institute of Photonic Technologies, Aston University, Birmingham, United Kingdom B4 7ET; 4 College of Precision Instrument and Optoelectronic Engineering, Tianjin University, Tianjin, 300072 China

## Abstract

Most overlooked challenges in ultrafast optical time stretch imaging (OTSI) are sacrificed spatial resolution and higher optical loss. These challenges are originated from optical diffraction devices used in OTSI, which encode image into spectra of ultrashort optical pulses. Conventional free-space diffraction gratings, as widely used in existing OTSI systems, suffer from several inherent drawbacks: limited diffraction efficiency in a non-Littrow configuration due to inherent zeroth-order reflection, high coupling loss between free-space gratings and optical fibers, bulky footprint, and more importantly, sacrificed imaging resolution due to non-full-aperture illumination for individual wavelengths. Here we report resolution-improved and diffraction-efficient OTSI using in-fiber diffraction for the first time to our knowledge. The key to overcome the existing challenges is a 45° tilted fiber grating (TFG), which serves as a compact in-fiber diffraction device offering improved diffraction efficiency (up to 97%), inherent compatibility with optical fibers, and improved imaging resolution owning to almost full-aperture illumination for all illumination wavelengths. 50 million frames per second imaging of fast moving object at 46 m/s with improved imaging resolution has been demonstrated. This conceptually new in-fiber diffraction design opens the way towards cost-effective, compact and high-resolution OTSI systems for image-based high-throughput detection and measurement.

## Introduction

Optical time stretch technology enables continuous ultrafast imaging at unprecedented speed of tens of million frames per second^1^. This new type of imaging technology, also known as serial time-encoded amplified microscopy (STEAM)^2^, overcomes fundamental trade-off between imaging speed and sensitivity in ultrafast imaging by combining unique space-spectrum-time mapping using spatial and chromatic dispersions, low noise optical amplification and high-speed single-pixel detection. Following its invention, optical time stretch imaging (OTSI) technology has enabled real-time monitoring of laser ablation process^2^, the discovery of optical rogue waves^3^, and high-throughput detection of rare circulating cancer cells in blood with unprecedented sensitivity^4^. It has also been successfully applied in ultrafast laser scanning^5,6^ and optical coherence tomography^7^. Great research efforts have been made to further the performance of OTSI with higher imaging speed^8^, better resolution^9,10^, and added phase contrast imaging capability^11^. In the meanwhile, the capability of OTSI, which was first implemented in the telecommunication wavelength band at around 1550 nm to take advantages of high-quality dispersive component in this band, has been made available in both shorter wavelength band^12–15^ for better imaging resolution and in longer wavelength range^16^ for deeper penetration through a scattering medium.

One remaining challenge in the emerging OTSI technology lies in its optical diffractive elements (ODEs), which is a key component in OTSI systems to achieve spectral encoding of the spatial information (image) based on space-to-spectrum mapping^17,18^. Most commonly used ODEs in existing OTSI systems are free-space ruled or holographic diffraction gratings, which however suffer from some inherent drawbacks. First, in order to achieve high angular dispersion for high-quality imaging, diffraction gratings with high groove density are usually required, which are however costly and bulky, and hinder miniaturization of the imaging system in real practice. Second, normal ruled or holographic diffraction gratings fall short in diffraction efficiency (usually up to 75%) due to the inherent zeroth-order reflection and non-Littrow configuration. Third, high coupling loss between free-space diffraction gratings and optical fibers or chirped fiber Bragg gratings, which provide chromatic dispersion for time stretching, is significant and worsens signal/image quality. In particular, many time stretch imaging systems are based on a reflection configuration^2,4–6^. Reflected light from the target is diffracted by the diffraction grating for the second time to be coupled back into optical fiber with the help of a fiber collimator. Use of a fiber collimator and double diffractions from the diffraction gratings lead to high coupling loss. More importantly, what is surprisingly overlooked in existing free-space diffraction grating based OTSI systems^2–14^ is the sacrificed imaging resolution (lower than diffraction limit) due to non-full-aperture illumination for individual wavelengths, which is resulted from large angular dispersion of free-space diffraction gratings. Most recently, full back aperture illumination for broadband light has been reported using free-space angular-chirp-enhanced delay (FACED)^15^. However, the FACED method uses a pair of bulky plane mirrors to gain chromatic and angular dispersion. Alignment-free, low intrinsic loss and system miniaturization are real challenges.

The motivation behind this work is to provide an optimal solution to all these challenges in OTSI. The key idea of our proposal is to take advantage of highly efficient in-fiber lateral diffraction, which is made possible by using a 45° tilted fiber grating (TFG)^19^. Considering that the TFG can be made long (serval cm), the interaction length for lateral diffraction is greatly enhanced and independent on the incident beam size. In addition, the 45° tilted grating structure removes cylindrical symmetry in the fiber core, making the 45° TFG highly polarization dependent with a significantly enhanced diffraction efficiency (up to 97%). We have previously demonstrated the utility of 45° TFG in spectrally-encoded imaging^20^. The use of 45° TFG in OTSI will completely eliminate the need for bulky free-space diffraction gratings for spectral encoding and avoid complex and lossy light coupling between dispersive fibers and free-space components. The 45° TFG can work in a reflection configuration due to reversibility of light path^21^, leading to further reduced coupling loss. This conceptually new design of all-fiber OTSI scheme significantly reduces the volume of the imaging system, improves diffraction efficiency, and enhances system stability. More importantly, the 45° TFG enables almost full-aperture illumination for all wavelengths due to its small angular dispersion value, leading to improved imaging resolution for a given imaging optics setup.

We present in this paper, for the first time to the best of our knowledge, resolution-improved and diffraction-efficient OTSI using a 45° TFG as an in-fiber diffraction grating. We fabricated the in-fiber diffraction grating device and experimentally investigated its utility in ultrafast time stretch imaging. As a proof of concept, 50 million frames per second imaging of fast moving object at 46 m/s with a large field of view of 0.7 mm and almost diffraction-limited resolution of 42.5 µm has been demonstrated. Superior performance in terms of imaging resolution compared to conventional free-space diffraction grating based OTSI has been verified via a side-by-side comparison, where the same focusing lens and same overall diffracted beam width have been selected. The developed in-fiber diffraction technique opens the way towards cost-effective, compact and high-resolution time stretch imaging for image-based high-throughput detection and measurement.

## Results

### Performance analysis of the 45° TFG

Compared with normal fiber Bragg gratings (FBGs)^22,23^, a TFG has periodical refractive index variation in axial direction, but its boundary surface of the varied index is tilted at a specific facet angle with respect to the fiber axis, endowing it with unique optical properties. Small angle TFGs, which excite light coupling from forward propagating core mode into backward propagating cladding and core modes, have been widely used in sensing applications^24–26^. A 45° TFG works in a dramatically different way. The forward propagating light is coupled into radiation modes owing to its largely tilted facet angle, leading to direct lateral diffraction into free space. Its wide operation window (several hundred nm) and wavelength dependent diffraction angle make the 45° TFG an excellent candidate for in-fiber lateral diffraction. In addition, with the circular symmetry of the fiber core being deliberately removed, the 45° TFG is highly polarization dependent with polarization dependent loss as high as 40 dB^27^, making it an ideal in-line fiber polarizer^28^. Owing to its unique light coupling features, 45° TFGs have found rich applications in optical spectrum analysis^29^, mode-locked lasers^30^, and optical coherence tomography^31^. Here we present the first comprehensive study and experimental demonstration of using a 45° TFG as a novel in-fiber diffraction device for OTSI. Since only the *s*-polarized light beam can be emitted out of the TFG, high diffraction efficiency can be guaranteed via proper polarization control of the incident light in the fiber core.

**Figure 1:**
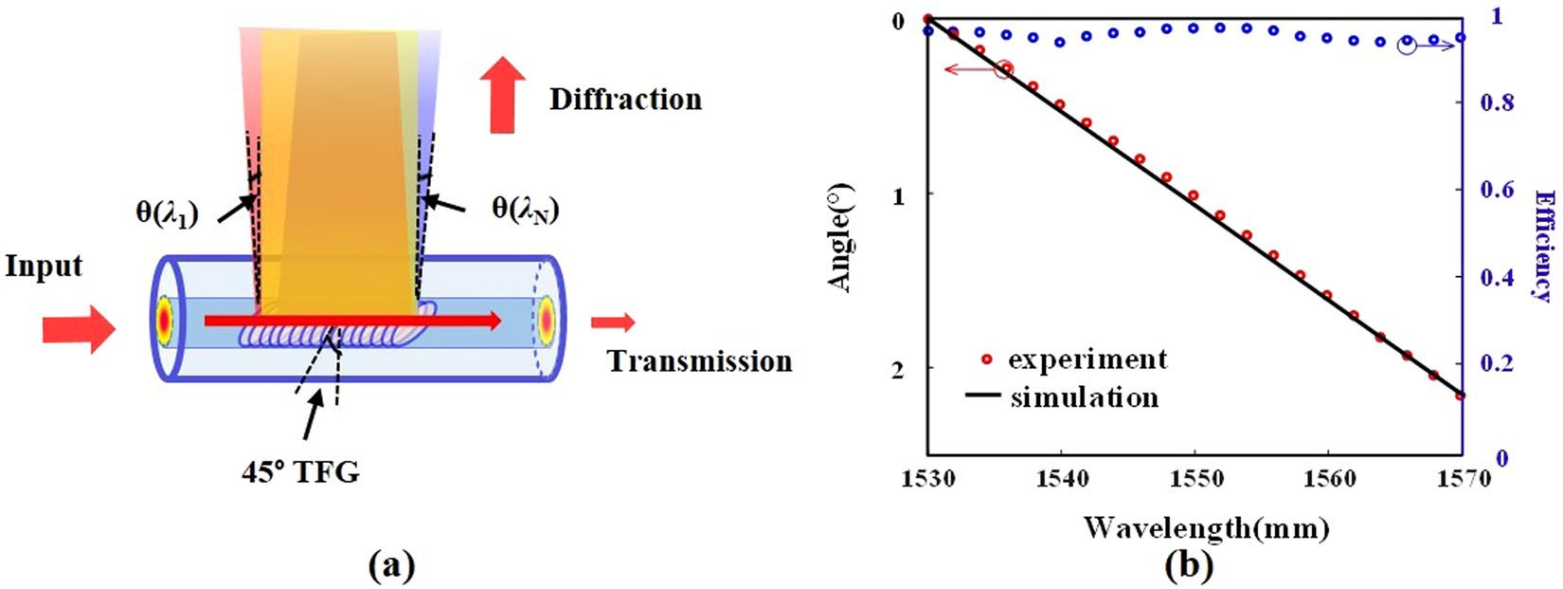
Concept of the 45° TFG. (**a**) Diagram showing the structure of the 45° TFG and its wavelength-dependent lateral diffraction. (**b**) The measured diffraction angle with respect to incident optical wavelength, showing angular dispersion of 0.054°/nm. Diffraction efficiency is up to 97% with an average value of 93.5% over a broad bandwidth from 1530 to 1570 nm.

The structure of a 45° TFG and its wavelength-dependent lateral diffraction is illustrated in Fig. 1(a). Forward propagating light in the fiber core is coupled into radiation modes at the 45° TFG. Taking into account the refraction at the interface between fiber cladding (refraction index of 1.44) and air, the scattering angle of emitted light out of TFG can be expressed as^32^

1 $$\sin [\alpha (\lambda )]=\frac{n\cdot (\cot \,\theta -\frac{n\cdot {\rm{\Lambda }}}{\lambda \cdot \,\sin \,\theta })}{\sqrt{1+{(\cot \theta -\frac{n\cdot {\rm{\Lambda }}}{\lambda \cdot \sin \theta })}^{2}}}$$

where $\alpha$ is the scattering angle off the fiber from the normal for a given optical wavelength *λ*, *n* is the refractive index of fiber core, *θ* is the tilt angle of the TFG, and $\Lambda$ is the grating period. For a 45° TFG with a grating period of 748 nm, the calculated angular dispersion at 1550 nm is 0.053°/nm.

A 24 mm long 45° TFG has been fabricated (see Methods) and its angular dispersion is measured. Figure 1(b) shows the measured scattering angle for broadband incident light from 1530 to 1570 nm. The experimental results indicate an angular dispersion of 0.054°/nm, which is in perfect agreement with the theoretical result according to (1). Note that there is an exponential decay in the diffracted intensity profile along the grating length. In all the following experiments, only the first 8 mm of the TFG, which contributes to the most diffraction power, is employed by blocking the remaining part of the grating. The fabricated TFG offers flat diffraction power (0.3 dB fluctuation) across 40 nm bandwidth. When the propagated light is tuned to be fully *s*-polarized, the diffraction efficiency is as high as 97% with an average value of 93.5% across the whole band (1530–1570 nm) as shown in Fig. 1(b), verifying its improved efficiency compared to conventional free-space diffraction gratings. As there is no Bragg reflection nor coupling into cladding modes in a 45° TFG, diffraction efficiency of the TFG is measured by comparing the diffracted and transmitted optical power^20^.

**Figure 2:**
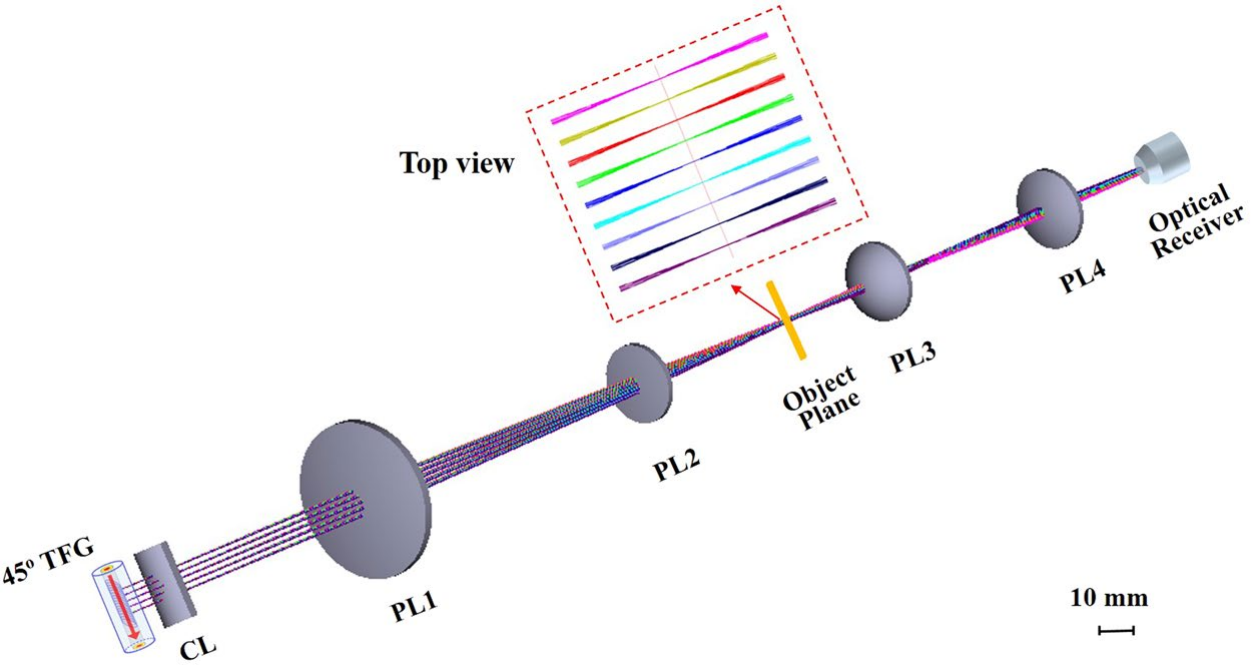
Simulation results. Ray-tracing simulation results using Zemax showing wavelength-to-space mapping for spectrally-encoded imaging with a shallow depth of field. The field of view is 1.4 mm. TFG, titled fiber grating; CL, cylindrical lens; PL, Plano-convex lens.

The utility of 45° TFG as an in-fiber diffraction grating for OTSI is first examined using a ray tracing software Zemax based on the TFG characteristics and actual experimental setup, with the results shown in Fig. 2. A cylindrical lens with 20 mm focal length is placed after the TFG to collimate the emitted optical beam in vertical direction. An imaging lens set consisting of two plano-convex lenses with focal lengths of 250 mm and 200 mm separated by 130 mm is used to focus different wavelengths of light (from 1530 to 1570 nm) into different spatial positions on the object plane. The inset shows a zoom-in top view of 1-D illumination beam near the object plane. Wavelength-to-space mapping has been achieved and different wavelengths of light are focused on the same object plane confirming a shallow depth of field.

### Time-stretch imaging using the 45° TFG

**Figure 3:**
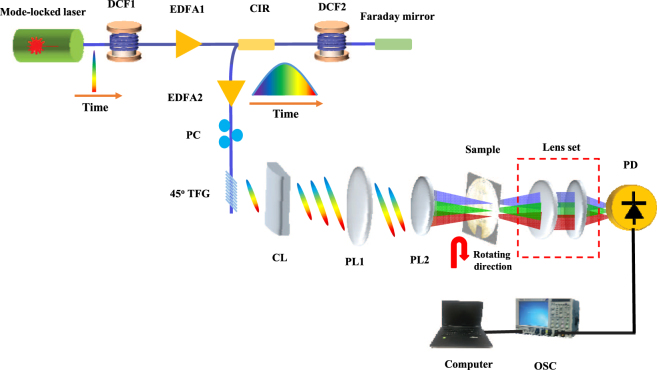
Schematic of the 45° TFG based ultrafast OTSI system. Ultrafast optical pulses from a mode-locked laser are largely stretched using dispersive compensating fibers (DCFs) with total dispersion of 1.03 ns/nm to achieve wavelength-to-time mapping, followed by optical amplification using two Erbium doped fiber amplifiers (EDFAs). Polarization state of time-stretched optical pulses are controlled by a polarization controller (PC) to ensure high diffraction efficiency of 45° TFG. A lens set focuses the illumination beam onto the object plane for wavelength-to-space mapping. The transmitted light is detected by a high-speed free-space photodetector with the help of a second lens set (See Methods). DCF: dispersive compensating fiber; EDFA: Erbium doped fiber amplifier; CIR: circulator; PC: polarization controller; TFG: tilted fiber grating; CL: cylindrical lens; PL: plano-convex lens; PD: photo-detector; OSC: oscilloscope.

The utility of the fabricated 45° TFG in ultrafast OTSI is experimentally demonstrated. We construct the imaging apparatus as shown in Fig. 3. A passively mode-locked fiber laser (Calmar Mendocino FP laser) produces a series of ultrashort optical pulses with full-width at half maximum (FWHM) pulse width of 800 fs, repetition rate of 50 MHz, FWHM spectral bandwidth of 8 nm, and average optical power of 15 mW. Optical pulses from the laser are largely stretched using dispersive compensating fibers (DCFs) with total dispersion of 1.03 ns/nm to achieve wavelength-to-time mapping, followed by optical amplification using two Erbium doped fiber amplifiers (EDFAs). Time-stretched and amplified optical pulses, with their polarization states properly controlled, are launched into the 45° TFG with effective length of 8 mm (see Methods), where light is scattered out of fiber into free space. A lens set (as depicted in Fig. 2) focuses the illumination beam onto the object plane. The imaging system is working in transmission mode. A second lens set is employed to collect the transmitted light and couple it into a high-speed single-pixel photodetector with an analog bandwidth of 12 GHz (see Methods). A high-speed oscilloscope (Tektronix DPO 72304DX) with a real-time sampling rate up to 100 GS/s captures and digitizes the time-encoded signal. All time-domain measurements are taken in single-shots without averaging. Image of the object is reconstructed in the digital domain.

**Figure 4:**
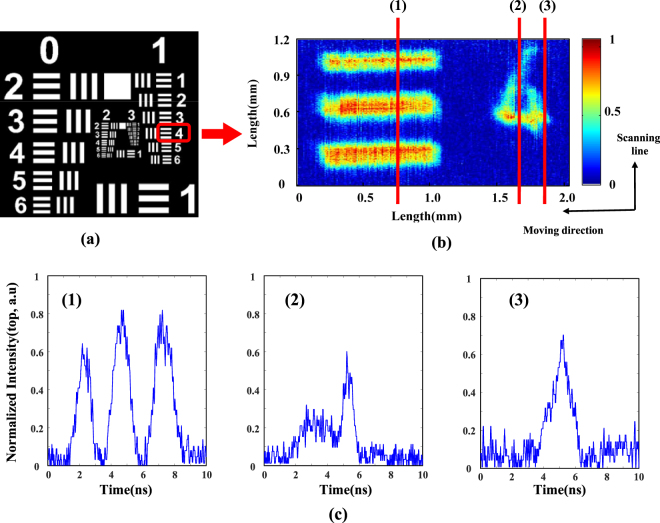
Imaging a stationary sample with the TFG-based OTSI. (**a**) CCD image of the resolution chart. (**b**) The reconstructed image of the selected part of the resolution chart. (**c**) Stretched and time-encoded optical waveforms corresponding to three different line scans in (**b**). Average optical power used is 15 mW.

The basic performance of the proposed TFG-based OTSI system is demonstrated by imaging a standard resolution test chart, with the results shown in Fig. 4. Element 4 in group 1 is imaged. The effective field of view of 1D line scan imaging is 1.2 mm in vertical direction. 2D image of the resolution chart was obtained by moving the chart in the horizontal direction with a fine step of 15 µm, with result shown in Fig. 4(b). Stretched and time encoded optical pulses corresponding to three different line scans are shown in Fig. 4(c) as well. To construct the image precisely, spatial and temporal calibration of the system is carried out (see Methods).

**Figure 5:**
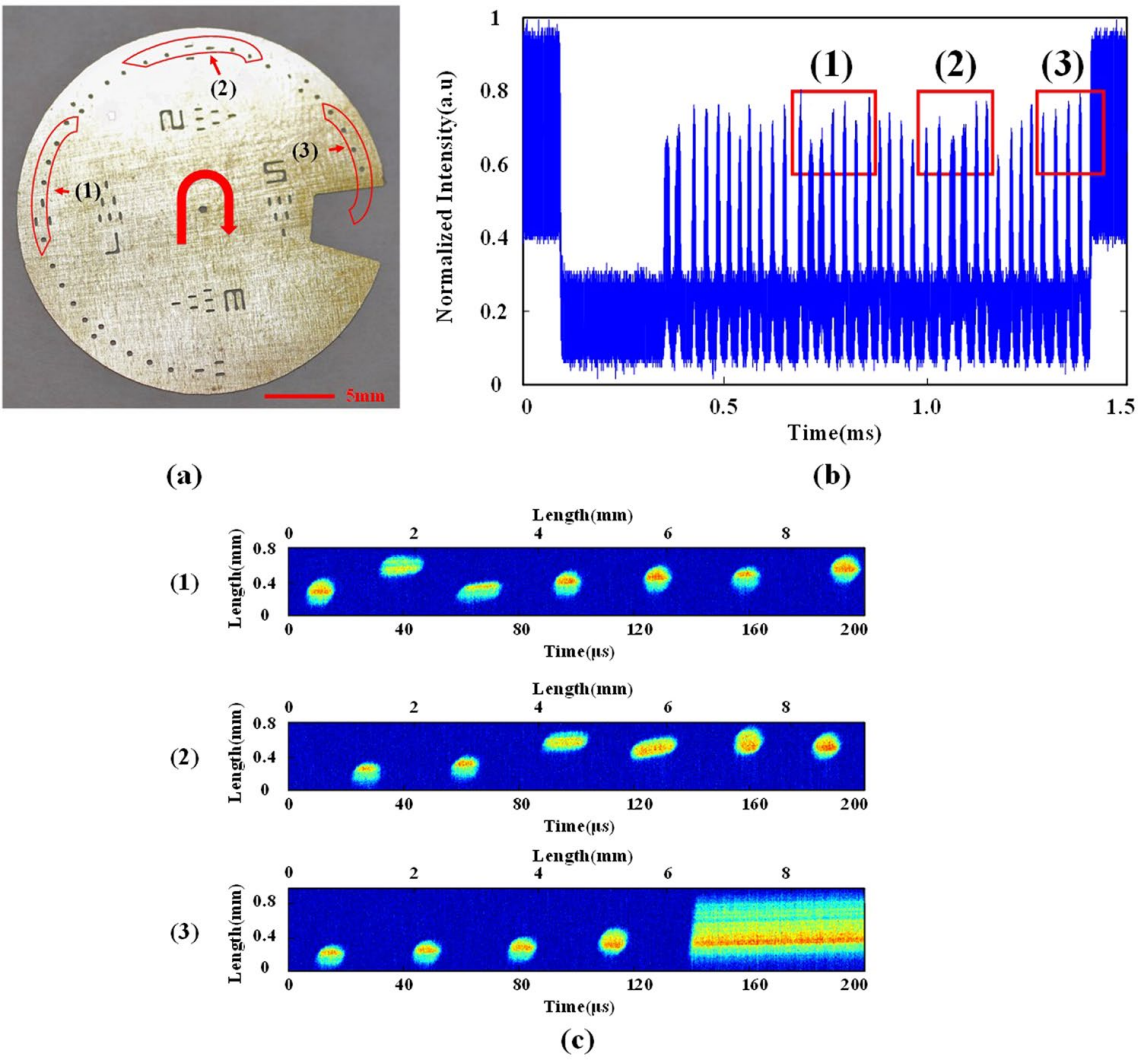
Imaging a fast moving object with the TFG-based OTSI. (**a**) CCD image of the one-inch sample with etched features. (**b**) The captured temporal waveforms representing the features in the sample. Waveforms corresponding to three groups of features have been highlighted with red frames. (**c**) The reconstructed images of the three groups of selected features in the fast spinning object at a line speed of 46 m/s. Average optical power used is 15 mW.

To demonstrate the ultrafast imaging capability of TFG-based OTSI system, we implemented real-time imaging of a fast-moving object. The sample is a custom-designed one-inch round metal disk with some etched features, as shown in Fig. 5(a). The metal disk, which is placed in the object plane, is spinning at a speed of 40,000 rpm driven by a mini motor (Coreless Motor 612), corresponding to a line speed of 46 m/s within the field-of-view range. The captured temporal waveform corresponding to a full rotation is plotted in Fig. 5(b). Three groups of different features in the sample, as highlighted by red frames, are represented by three groups of pulse bursts in red frames of Fig. 5(b). Each pulse in the bursts performs a single-shot line scanning of the spinning sample. The reconstructed images of selected features in the fast spinning object are shown on Fig. 5(c). Small circular holes, longer slots and a big gap have been clearly identified. Each of the images consists of 10,000 line scans. Each of line scans has a frame time of 20 ns and the whole image takes 200 μs. The actual field of view of each 2D image is 0.7 by 9.2 mm.

### Improved imaging resolution

**Figure 6:**
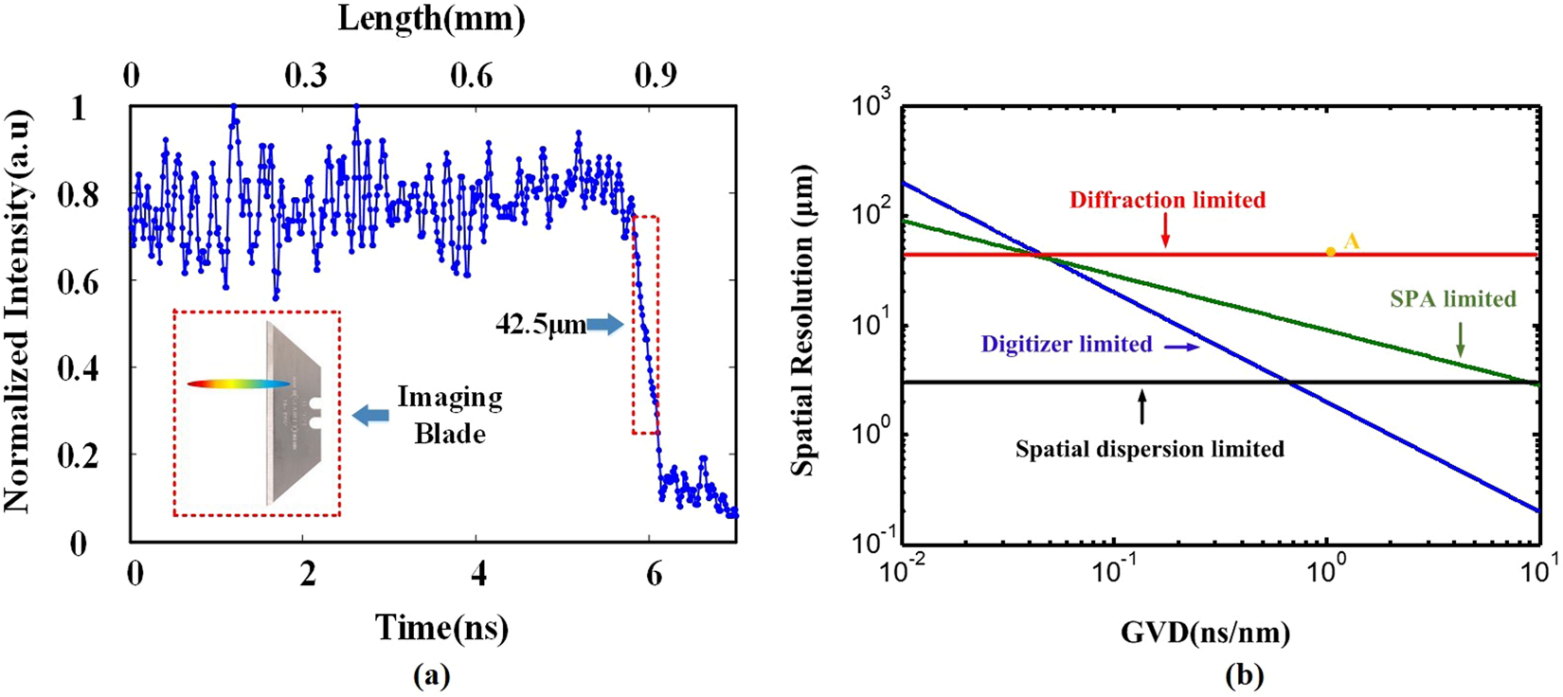
Spatial resolution of the TFG-based OTSI system. (**a**) Measurement of lateral resolution of the proposed TFG-based OTSI system based on point spread function by using a sharp blade. (**b**) Spatial resolution analysis of our proposed TFG-based imaging system. Experimental results show that the resolution of TFG-based OTSI system is 42.5 µm, which is very close to the diffraction limit resolution. Average optical power used is 15 mW.

**Figure 7:**
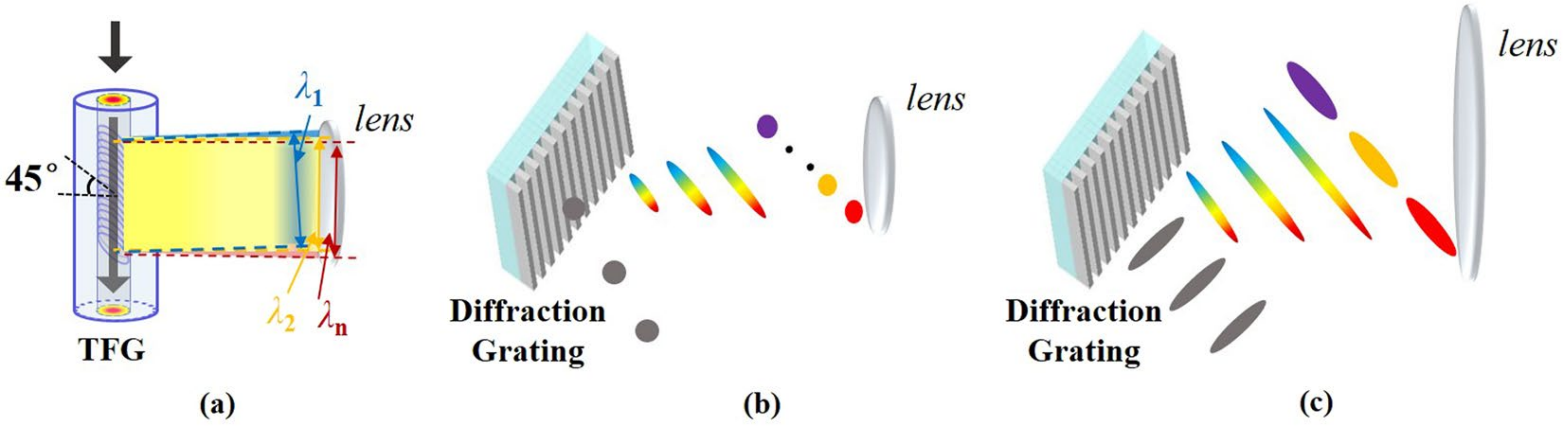
Principle of improved spatial resolution in TFG-based OTSI system. (**a**) The 45° TFG-based OTSI system offers improved imaging resolution due to almost full-aperture illumination for all illumination wavelengths. (**b**) Conventional OTSI systems use free space diffraction gratings and hence suffers from sacrificed imaging resolution if using a same imaging lens as diffracted beams with different wavelengths are largely separated and full-aperture illumination is not fulfilled for individual wavelengths. (**c**) In conventional OTSI systems, if the incident light has a large beam width, a very large focusing lens is needed to cover the whole diffracted illumination beam, which is bulky and expensive.

The spatial resolution of our proposed TFG-based OTSI system is estimated based on point spread function measurement using a sharp edge blade. A lateral resolution of 42.5 µm is obtained, as shown in Fig. 6(a). The spatial resolution of our proposed system is further analyzed. Spatial resolution of a OTSI system is determined by various factors^33^: the spectral resolution of the spatial disperser (spatial dispersion limited spatial resolution), focusing power of the plano-convex lenses (diffraction limited spatial resolution), the spectral resolution imposed by dispersion-induced time stretch through stationary-phase-approximation (SPA) (SPA limited spatial resolution), and the temporal resolution of the digitizer (digitizer limited spatial resolution). Figure 6(b) shows the calculated spatial resolution limited by different factors with respect to chromatic dispersion. The diffraction limited spatial resolution is calculated to be 41.7 μm according to the system parameters in our experiments. Owing to the extremely high grating groove density in the 45° TFG, the spatial dispersion limited resolution is as high as 3.2 μm. Considering that the group velocity dispersion (GVD) in our imaging system is 1.03 ns/nm, the measured spatial resolution is mapped into Fig. 6(b) as point A (42.5 µm), which is very close to the diffraction limited spatial resolution of our imaging system. Improved spatial resolution is due to the fact that the 45° TFG has much smaller angular dispersion value than a normal free-space diffraction grating and the whole beam width of diffracted light is similar to that of initial illumination beam. Almost full-aperture illumination is hence achieved for all wavelengths, as illustrated in Fig. 7(a). In order to achieve microscopic imaging, spatial resolution of our system can be improved by using a high-quality objective lens. Capability of improving imaging resolution can be preserved. Note that exponential decay of diffracted intensity over TFG may slightly degrade the imaging resolution compared to an ideal Gaussian beam.

**Figure 8:**
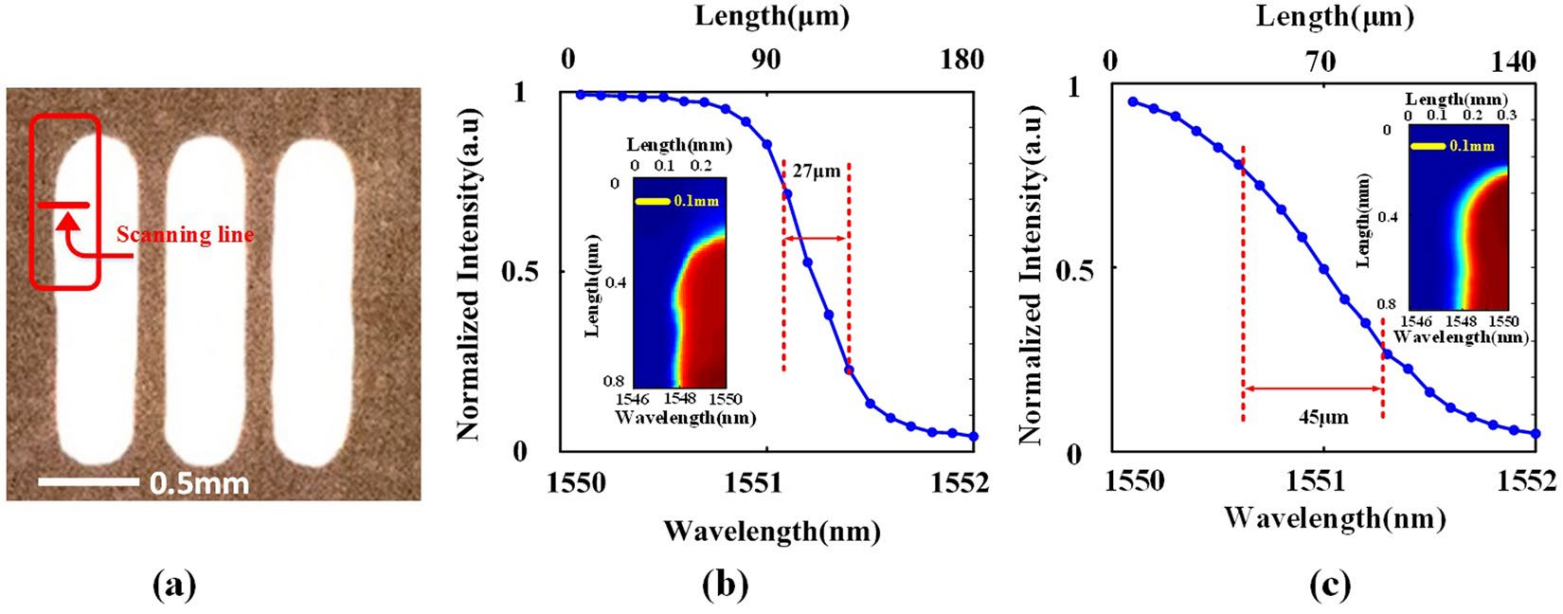
Comparison of spatial resolution in the proposed TFG-based and conventional OTSI systems. (**a**) CCD image of the sample used in comparison experiments (Imaging area is within the red frame). (**b**) Point spread function measurement and the reconstructed image using the TFG-based system. (**c**) Measurement results using conventional free-space diffraction gratings. Average optical power used is 15 mW.

Different from our proposed TFG-based system, conventional OTSI systems suffer from sacrificed spatial resolution if a same imaging lens is used, which is usually poorer than the diffraction limit. This is because a free-space diffraction grating has large angular dispersion value resulted from its small groove spacing and the diffracted optical beams with different wavelengths are largely separated. Therefore, each individual illumination wavelength does not fulfil the full-aperture illumination condition required for high-resolution imaging, as illustrated in Fig. 7(b). In addition, for a big initial illumination beam, a very large focusing lens is needed to cover the whole diffracted illumination beam, which is bulky and expensive. To compare the imaging resolution, two time stretch imaging experiments are carried out using a conventional free-space diffraction grating (Thorlabs GR25–0616) and the 45° TFG respectively. To ensure a fair side-by-side comparison, the spectral bandwidth and beam size of the illumination light out of the diffraction devices (TFG and free-space diffraction grating) remain identical in two systems and the same focusing lenses are used. Figure 8 shows the comparison results. The object is a thin metal plate with three etched slots, as shown in Fig. 8(a). Upper left corner of the first slot is imaged using two systems. The spatial resolutions achieved by our proposed and the conventional systems are characterized as 27 and 45 µm, respectively, based on point spread function measurement, as shown in Figs. 8(b) and (c). The reconstructed images are also presented. Superior performance of our proposed TFG-based approach in achieving higher imaging resolution has been evident.

## Discussion

In summary, we proposed and experimentally demonstrated the first use of a 45° TFG in ultrafast OTSI system enabling highly efficient ultrafast time stretch imaging with improved imaging resolution. The 45° TFG is presented as an in-fiber diffraction device to replace bulky and lossy free-space diffraction gratings in conventional OTSI systems. As the 45° TFG is inherently compatible with optical fibers that provide chromatic dispersion for time stretching, no coupling loss between free-space and fiber optics will occur. This new proposal simplifies the OTSI system, reduces system volume and cost, and promotes higher diffraction-efficiency, and better stability due to alignment-free optical diffraction. A 45° TFG was fabricated with an angle dispersion of 0.054°/nm and an enhanced diffraction efficiency up to 97% for equivalent grating length of 8 mm. Utility of the 45° TFG in ultrafast time stretch imaging has been verified by proof-of-concept demonstrations, where a fast moving object at 46 m/s was imaged at frame rate of 50 million frames per second with a large field of view of 0.7 mm. Improved spatial resolution has been achieved due to the fact that the TFG enables almost full-aperture illumination for all the wavelengths. A side-by-side comparison between the proposed in-fiber diffraction approach and the conventional free-space diffraction grating based OTSI systems have been carried out to evidence the superior performance of our approach. This conceptually new in-fiber diffraction based OTSI scheme opens the way towards cost-effective, compact and high-resolution ultrafast imaging systems for image-based high-throughput detection and measurement.

## Methods

### Fabrication of the 45° TFG

A 45° TFG with a period of 748 nm was fabricated to function as an efficient in-fiber diffractive element in the proposed imaging system. Considering the incident light propagates inside fiber core, the equivalent groove period is the product of refractive index and the actual period, which is in the order of the optical wavelength. The TFG was written directly into a standard telecom single-mode fiber (SMF-28) using the standard scanning phase mask technique with continuous-wave UV-light at 244 nm. To achieve the required 45° slanted grating fringes, the phase mask was rotated by 33.3°. The fabricated TFG is 24 mm long, ensuring high efficiency of diffraction. There is an exponential decay for the diffracted intensity profile along the propagation direction in TFG^20^. In the imaging experiments, only the first 8 mm of the TFG, which contributes to the most diffraction power, was used by blocking the remaining part of the grating.

### Light collection at the photodetector

The imaging system is working in a transmission configuration. Diffracted light beam propagated through the object is not properly collimated (light with different wavelengths will have different propagation angles). To focus the light beam onto the high-speed free-space photodetector with a small sensitive area of 50 µm by 50 µm, two stages of light collection were arranged. In the first stage, a plano-convex lens with focal length of 30 mm is used to couple the light into a large numerical aperture (NA) multimode fiber via a fiber collimator. Here a single mode fiber is not required because chromatic dispersion is already provided before light is emitted from the TFG. Secondly, beam size of the output light from the multimode fiber through a second collimator is reduced by 4 times using a telescope to match the sensor size of the high-speed photo-detector for efficient light detection.

### Spectral and temporal calibration

The space-to-spectrum conversion is achieved based on the use of diffraction device. This mapping relationship is first calibrated by sweeping the wavelength of a tunable continuous-wave laser and measuring the displacement of the focused laser spot on the object plane using an infrared beam profiler. This is characterized as 1 nm (spectrum) to 0.1 mm (space). The spectrum-to-time conversion is based on dispersion-induced time stretch. As significant amount of dispersion is required, higher-order dispersion (dispersion slope) may have impact on the spectrum to time mapping process. Wavelength-dependent total group delay dispersion (GVD) of the DCF used is characterized accurately by comparing interferograms in the wavelength domain and in the time domain^34^. The nominal GVD value is evaluated as 1030 ps/nm.

### Data Availability

The authors are willing to share their raw data by uploading data files to an open website if the manuscript is accepted for publication.
